# A Pharmacokinetic–Pharmacodynamic Study of Protosappanoside D, a Component Derived from *Biancaea decapetala* Extracts, for Its Anti-Inflammatory Effects

**DOI:** 10.3390/ijms26083694

**Published:** 2025-04-14

**Authors:** Zuying Zhou, Yang Zhou, Pu Wang, Ting Zhou, Mingyan Chi, Yueting Li, Meng Zhou, Shuai Yang, Aimin Wang, Lin Zheng, Yong Huang

**Affiliations:** 1 State Key Laboratory of Discovery and Utilization of Functional Components in Traditional Chinese Medicine, Guizhou Provincial Key Laboratory of Pharmaceutics, Guizhou Medical University, Guiyang 550004, China; zu_ing@163.cn (Z.Z.); zy225300@163.com (Y.Z.); z_tillie@163.com (T.Z.); nhwslyt@163.com (Y.L.); ys101352@126.com (S.Y.); 2 School of Pharmaceutical Sciences, Guizhou Medical University, 4 Beijing Road, Guiyang 550004, China; wangpu@gmc.edu.cn (P.W.); chimingyan@gmc.edu.cn (M.C.); 3 National Engineering Research Center of Miao’s Medicines, Engineering Research Center for the Development and Application of Ethnic Medicine and TCM (Ministry of Education), Guizhou Medical University, Guiyang 550004, China; gmu_mengzhou@163.com (M.Z.); gywam100@163.com (A.W.)

**Keywords:** *Biancaea decapetala*, Protosappanoside D, rheumatoid arthritis, pharmacokinetics–pharmacodynamics model

## Abstract

*Biancaea decapetala* (Roth) O. Deg. (Fabaceae), traditionally used by the Hmong people to treat rheumatoid arthritis (RA), has not been extensively studied for the correlation between its anti-inflammatory activity and its active components. Protosappanoside D (PTD), a new component, has been isolated for the first time from the extract of *Biancaea decapetala*. This study focused on the anti-arthritic and anti-inflammatory effects of *Biancaea decapetala* extracts (BDE) and PTD, along with their pharmacokinetic–pharmacodynamic (PK-PD) analysis. In the adjuvant-induced arthritis (AA) rat model, HE staining and cytokine assays showed that BDE alleviated joint damage and reduced inflammatory cytokines, similar to the positive control. In the LPS-induced inflammatory cell model, both BDE and PTD demonstrated anti-inflammatory effects by inhibiting the secretion of inflammatory factors. A PK-PD analysis of BDE in AA rats and inflammatory cells, as well as an analysis of PTD as a monomer, was conducted. The results indicated that PTD had different regulatory effects on cytokines like TNF-*α*, with a certain lag and sustained effects. These findings suggest the potential of BDE and PTD as treatments for rheumatoid arthritis, though further in vivo studies and clinical trials are needed.

## 1. Introduction

The pharmacokinetic–pharmacodynamic (PK-PD) model is an essential tool for studying traditional Chinese medicines (TCMs), linking drug metabolism with therapeutic effects [1,2]. By integrating dynamic pharmacokinetic changes with measures of drug efficacy, this model provides a comprehensive framework for understanding dosage–effect relationships [3,4]. However, challenges remain in developing PK-PD models for TCMs due to their complex mechanisms in disease treatment. The key issues include the following: (1) an excessive focus on pharmacokinetic studies in healthy organisms, with insufficient attention to the significance of PK-PD model research under disease conditions; and (2) a predominant reliance on whole-animal studies for TCM PK-PD models, with inadequate incorporation of cellular pharmacokinetics principles. Since some TCM bioactive substances target intracellular sites, this limitation results in incomplete research findings.

Cellular pharmacokinetics, an extension of traditional pharmacokinetics, has recently gained attention for its ability to analyze drug absorption, distribution, metabolism, and elimination at the cellular level [5,6]. By integrating mathematical models, this approach clarifies drug interactions with intracellular targets and enhances our understanding of drug efficacy. Unlike traditional pharmacokinetics, which primarily examines macroscopic processes, cellular pharmacokinetics provides deeper insights into microscopic drug metabolism dynamics [7]. Despite its broad application in evaluating anticancer drugs and antibiotics, cellular pharmacokinetics has been less utilized in TCM research [8]. To expand its application, Academician Guangji Wang of the Chinese Academy of Engineering has pioneered the use of cellular pharmacokinetics theory in TCM research, achieving significant results [9]. Consequently, while classical pharmacokinetic studies may not adequately address the discrepancies between pharmacokinetics and pharmacodynamics in TCMs, employing cellular pharmacokinetics offers a novel perspective.

*Biancaea decapetala* (*B. decapetala*), a medicinal plant from the Fabaceae family, is known for its detoxifying and anti-inflammatory properties [10]. Current applications include drugs, such as Qingbi Tongluo Yaojiu and the Yun Shi Gan Mao Mixture, highlighting its therapeutic potential [11]. Recent research on *B. decapetala* has predominantly focused on its chemical constituents and pharmacological activities, identifying key active components, such as terpenoids, flavonoids, and coumarins [12]. Notably, Protosappanoside D (PTD), a newly isolated component from *B. decapetala*, has shown significant promise [13]. PTD, structurally classified as a homoisoflavone along with its aglycone protosappanin B (PTB) and 3-deoxysappanchalcone (3-DSC), has demonstrated substantial anti-inflammatory effects [14]. Studies have indicated that PTD inhibits pro-inflammatory cytokine secretion in LPS-stimulated RAW264.7 cells, sharing a similar core molecular structure with PTB. Preliminary serum pharmacochemistry studies on rats administered *B. decapetala* extracts identified PTD as one of the primary active ingredients [13]. Despite extensive studies on the pharmacological properties and chemical composition of *B. decapetala*, further investigation into the correlation between its anti-inflammatory activity and active components, particularly PTD, is warranted. This study seeks to clarify the therapeutic material basis of *B. decapetala*, investigating PTD’s potential as a treatment for inflammatory diseases, such as rheumatoid arthritis.

In this study, we conducted PK-PD research concurrently at both the animal and cellular levels, focusing on the bioactive component PTD. The concentration of PTD was assessed in adjuvant-induced arthritis (AA) model rats and inflammatory cells as a pharmacokinetic indicator. Additionally, TNF-*α*, IL-1*β*, IL-6, and RF were used as in vivo pharmacodynamic indicators, while NO, TNF-*α*, and IL-6 served as in vitro pharmacodynamic indicators. A PK-PD model for *B. decapetala* was constructed using WinNonLin software (version 8.2) to analyze the relationship between anti-inflammatory effects, drug concentrations, and time in rats and cells, thereby fitting the PK-PD parameters. This approach established the dose-response relationship for the anti-inflammatory effects of PTD under inflammatory conditions, elucidating the therapeutic basis of *B. decapetala*. Furthermore, we conducted an in-depth PK-PD analysis of PTD as a standalone entity in inflammatory cells, providing deeper insights into its efficacy and mechanism of action.

## 2. Results

### 2.1. Composition Analysis of BDE and Its Medicated Plasma of Model Animals

**Figure 1:**
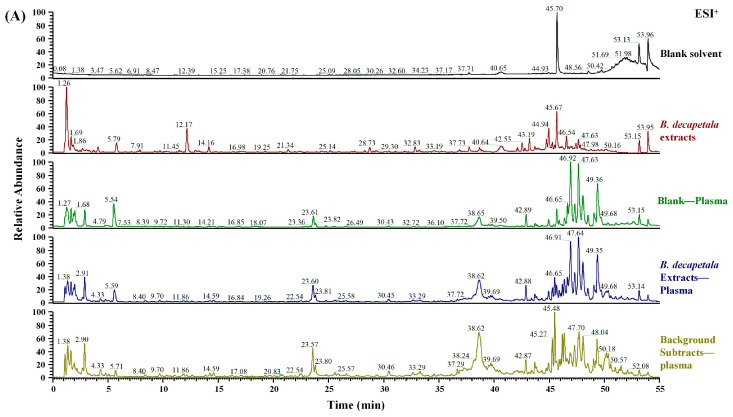

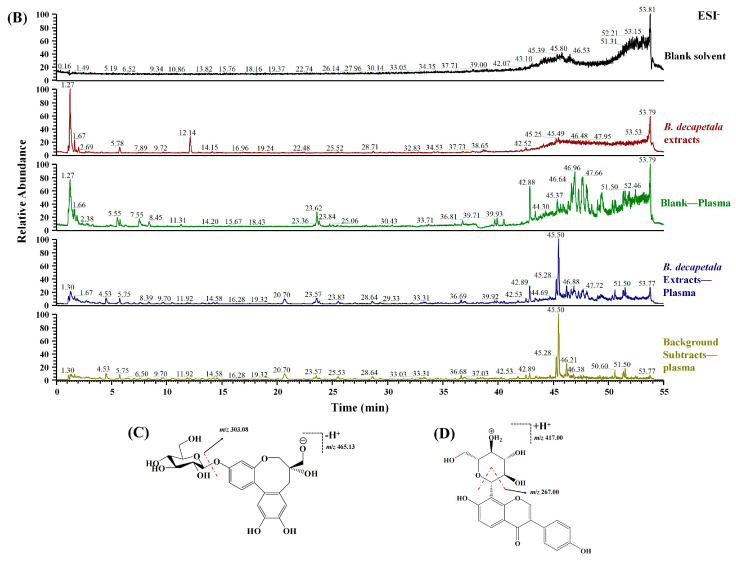
Total ion chromatograms of the *Biancaea decapetala* extracts and plasma samples collected after drug administration in model rats: (**A**) positive ion mode; (**B**) negative ion mode, and chemical structure of (**C**) PTD and (**D**) Puerarin.

**Figure 2:**
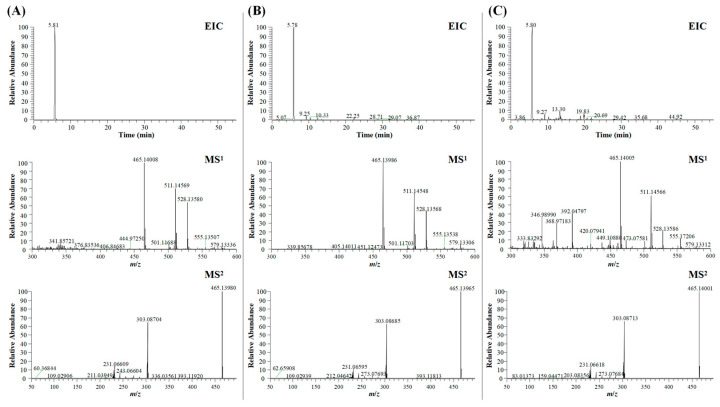
Extracted-ion chromatograms (EICs) and primary and secondary mass spectra of Protosappanoside D in (**A**) reference substances, (**B**) BDE, and (**C**) plasma samples. MS^1^ refers to primary fragment ions, MS^2^ refers to secondary fragment ions.

UHPLC/Q Exactive Plus Orbitrap HRMS was employed to acquire the total ion chromatograms (TIC, as presented in Figure 1) of the extracts from BDE and the plasma samples collected post-drug administration in model rats. The identification of chemical components was carried out using the Thermo Compound Discoverer 3.2. In total, 63 compounds were identified within BDE, and their identities were verified by a comparison with either controls or the standards database. These compounds encompassed diverse types, including phenolic acids, coumarins, flavones, alkaloids, and others. Notably, PTD, Protosappanin B, lsoliquiritigenin, and a total of 29 other components were found to be absorbed into the bloodstream. The detailed identification information of these compounds is provided in Table S1. As shown in Figure 2, the extracted ion chromatogram (EIC) shows chromatographic peaks around 5.8 min in all three samples, indicating they belong to the same compound. The MS^1^ spectra reveal a primary ion peak at *m*/*z* 465.14008, accompanied by *m*/*z* 511.14569, with slight variations across different samples. The MS^2^ spectra further analyze ion fragmentation, showing characteristic peaks at *m*/*z* 303.08704 in all three samples, though their relative intensities and distributions differ, reflecting structural variations between the samples. This figure systematically illustrates the chromatographic and mass spectrometric features of different samples, providing a foundation for further analysis.

### 2.2. Method Validation for PTD Quantification in Plasma and Cell Samples

The UPLC-MS/MS method for quantifying PTD in plasma and cell samples was validated according to the Chinese Pharmacopoeia guidelines, assessing specificity, linearity, lower limit of quantification (LLOQ), accuracy, precision, recovery, matrix effects, and stability. For plasma samples, specificity was confirmed by comparing the chromatograms of blank plasma, blank plasma spiked with PTD (LLOQ) and puerarin, and actual plasma samples, showing no interference from endogenous substances (Figure S1). The calibration curves for PTD demonstrated excellent linearity (R^2^ > 0.999) with an LLOQ of 93.94 ng/mL (Table S2). The intra- and inter-day precision (RSD < 15%) and accuracy (RE within ± 15%) met the acceptance criteria, while the recovery rates (97.11–108.90%) and matrix effects (95.98–100.48%) indicated minimal interference (Tables S3 and S4). The stability tests under room temperature, refrigeration, and freeze–thaw cycles confirmed analyte stability (RSD within ± 15%, Table S5).

For cell samples, the method was validated for PTD, with no endogenous interference observed in MRM chromatograms (Figure S2). The regression equation for PTD in cell lysates was Y = 0.006X − 0.0430, with the intra- and inter-day precision < 15% and accuracy ranging from 85% to 115% (Tables S6 and S7). The recovery, matrix effects, and stability also met the acceptance criteria (RSD < 15%, Tables S8 and S9). These results collectively demonstrate the method’s reliability for pharmacokinetic studies in both plasma and cell matrices.

### 2.3. Validation of the AA Rat Model and Anti-Arthritic Effects of BDE

**Figure 3:**
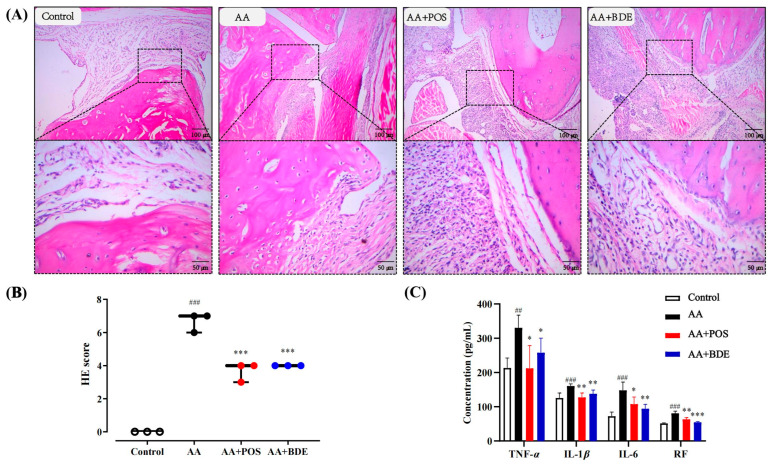
Results of in vivo AA model validation and the effects of BDE (4.5 g/kg, twice daily) and POS (102 mg/kg, twice daily) on it. Hematoxylin–eosin staining of ankle joints in rats of different groups with different magnification (**A**). Comparison of HE scores among groups was conducted using nonparametric test (**B**). Levels of TNF-*α*, IL-1*β*, IL-6, and RF in plasma of rats in each group (**C**). Comparison between groups was conducted using one-way analysis of variance (ANOVA). Data were presented as mean ± SD (*n* = 6); * *p* < 0.05, ** *p* < 0.01, *** *p* < 0.001 compared with control group; ^##^ *p* < 0.01, ^###^ *p* < 0.001 compared with AA group.

The histopathological analysis using HE staining revealed distinct morphological changes in the joints of AA model rats. The control group exhibited normal joint structures with a single layer of synovial cells and no signs of inflammation. In contrast, the AA group showed severe synovial hyperplasia, fibroblast proliferation, fibrous tissue formation, and extensive inflammatory cell infiltration, consistent with rheumatoid arthritis pathology. Treatment with tripterygium glycosides and BDE significantly alleviated these pathological changes, reducing synovial hyperplasia and inflammatory cell infiltration (Figure 3A). These findings suggest that BDE effectively attenuates joint damage in AA rats, likely through its anti-inflammatory and immunomodulatory properties.

The cytokine assays demonstrated that plasma levels of TNF-*α*, IL-1*β*, IL-6, and RF were significantly elevated in the AA group compared to the control group (*p* < 0.01), confirming successful AA model induction. Both BDE and POS treatments markedly reduced these inflammatory markers (*p* < 0.01), indicating their potent anti-arthritic effects (Figure 3B). The reduction in pro-inflammatory cytokines, particularly TNF-*α* and IL-6, aligns with the known role of these mediators in driving synovial inflammation and joint destruction in rheumatoid arthritis. The observed decrease in RF levels further supports the potential of BDE to modulate autoimmune responses. These results collectively suggest that BDE exerts anti-rheumatoid arthritis effects by suppressing inflammatory cytokine production and mitigating joint pathology. The efficacy of BDE, which was comparable to the AA + POS group (tripterygium glycosides), highlights its potential as a therapeutic agent for rheumatoid arthritis. These findings provide a robust foundation for subsequent PK-PD studies, which will further elucidate the PK behavior and PD mechanisms of BDE in AA rats.

### 2.4. Validation of the LPS-Induced Inflammatory Cell Model and Anti-Inflammatory Effects of BDE and PTD

**Figure 4:**
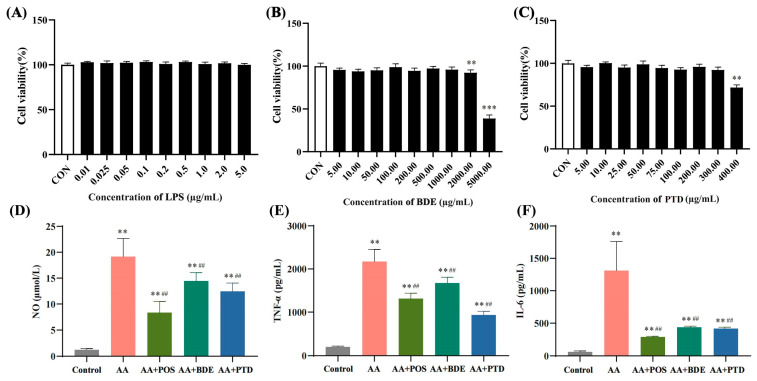
Results of in vitro inflammation model validation. Survival rate of RAW264.7 cells of LPS (**A**), BDE (**B**), and PTD (**C**) at different concentrations for 24 h, and accumulation of NO (**D**), TNF-α (**E**), and IF-6 (**F**) in LPS-induced RAW264.7 cells for 24 h. Comparison between groups was conducted using ANOVA. Data were presented as mean ± SD (*n* = 6); ** *p* < 0.01, *** *p* < 0.001 compared with control group; ^##^ *p* < 0.01 compared with model group.

The cells were treated with various concentrations of LPS. Figure 4A shows that after 24 h, the cell survival rate was unaffected. Based on this, 0.5 μg/mL of LPS was used for the LPS-induced inflammatory cell model. According to Figure 4B,C, cell viability remained above 90% across BDE concentrations from 0 to 1000 μg/mL and PTD concentrations from 0 to 400 µmol/L, indicating negligible cytotoxic effects. Consequently, 800 μg/mL of BDE and 200 µmol/L of PTD were chosen for further pharmacokinetic studies.

In the result of the evaluation of LPS-induced inflammatory cell model, cytokine determination results were shown in Figure 4D–F. Compared with the control group, the supernatant levels of NO, TNF-*α*, and IL-6 in the AA group were significantly increased (*p* < 0.01). Compared to the AA group, the levels of the four inflammatory factors in the cell supernatants of the AA + BDE, AA + POS, and PTD groups were significantly reduced (*p* < 0.01). This indicates that both the AA + BDE, AA + POS, and PTD groups all had a marked inhibitory effect on the secretion of TNF-*α*, IL-1*β*, IL-6, and RF in cells. In the LPS-induced validation cell model, an increase in indicators, such as NO, TNF-*α*, and IL-6, typically indicates the successful activation of inflammatory pathways in response to LPS stimulation, which establishes a foundation for further exploration into the PK-PD relationships at the cellular level.

### 2.5. PK-PD Analysis of PTD Within BDE in AA Rat Model

**Figure 5:**
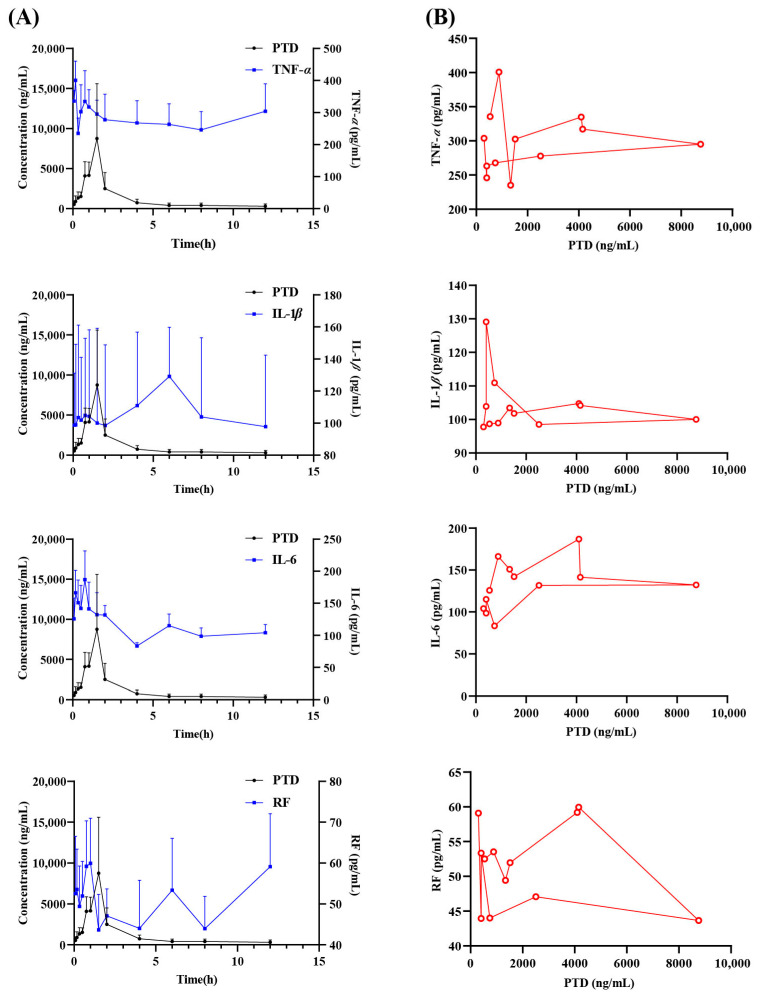
The concentration–time–effect curves (**A**) and concentration–effect curves (**B**) of PTD with TNF-*α*, IL-1*β*, IL-6, and RF in the plasma of AA model rats after the administration of BDE. The red lines represent the relationship between the drug concentration in vivo and the pharmacodynamic index concentration in vivo.

Using TNF-*α*, IL-1*β*, IL-6, and RF as PD indicators, we analyzed the changes in the plasma concentration–time–effect profiles in AA rats using the Phoenix modeling tool (Figure 5A). The effect–concentration curves were generated with a semi-compartmental modeling approach (Figure 5B), and the detailed values for PK and PD indicators over time are provided in Table S10. The pharmacokinetic parameters of PTD are provided in Table S11. In the PK and PD correlation analysis, a counterclockwise hysteresis loop typically indicates that the effect of a drug increases over time at a given concentration, whereas a clockwise hysteresis loop signifies a decrease in effect. Generally, a counterclockwise hysteresis loop is attributed to the presence of an effect compartment or the indirect actions of the drug, such as the pharmacological effects of its metabolites, while a clockwise hysteresis loop is often due to an acute tolerance or the formation of inhibitory metabolites. The effect–concentration curves illustrated that the PTD concentrations exhibited a clockwise hysteresis loop relative to TNF-*α*, IL-1*β*, IL-6, and RF. The lag between drug concentrations and inflammatory cytokine levels may result from the delay in drug distribution from the circulatory system to the joint cavity.

Utilizing a sigmoid Imax model, the corresponding PD values for PTD can be calculated from the plasma concentrations, and the corresponding blood drug concentrations in AA rats can also be calculated from the PD values. The PK-PD parameters are summarized in Table 1. IC50 is the drug concentration at which the effect reaches half of its maximum inhibition. This value reflects the drug’s potency and specificity: a smaller IC50 indicates greater potency and specificity, while a larger IC50 suggests lower potency and specificity. The K_e0_ parameter, representing the first-order rate constant for drug elimination from the effect compartment, characterizes the time course between the drug concentration and effect. As shown in Table 1, the K_e0_ value of TNF-*α* for PTD was 3.4 × 10^−4^, suggesting a moderate delay in modulating TNF-*α*, implicating TNF-α as a primary target for PTD’s anti-rheumatoid arthritis effects in vivo. Similarly, IL-1*β* and RF were identified as the main targets for PTD.

### 2.6. PK-PD Analysis of PTD Within BDE in LPS-Induced Inflammation Cell Model

**Figure 6:**
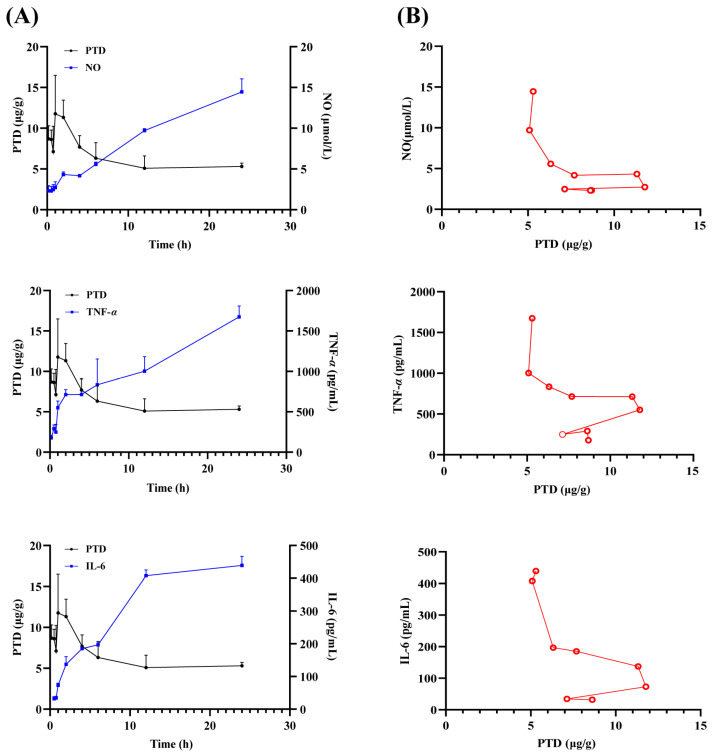
Concentration–time–effect curves (**A**) and concentration–effect curves (**B**) of PTD with NO, TNF-*α*, and IL-6 in inflammatory cells after administration of BDE. The red lines represent the relationship between the drug concentration and the pharmacodynamic index concentration.

For TCMs with intracellular targets, the free drug concentration within target cells is critical for pharmacological effects. Furthermore, intracellular pharmacokinetics are closely linked to drug efficacy, and in vitro PK-PD models provide insights into the concentration-time profiles and drug effects at the cellular level. Using NO, TNF-*α*, and IL-6 as PD indicators, we analyzed the changes in drug concentration-time-effect profiles in LPS-induced inflammation cell models using the Phoenix modeling tool (Figure 6A) and obtained the PK-PD parameters (Table 2). Effect-concentration curves were generated with a semi-compartmental modeling approach (Figure 6B), and detailed values for PK and PD indicators over time are provided in Table S12. The pharmacokinetic parameters of PTD are provided in Table S13.The concentration-time-effect profiles indicated that peak concentrations of PTD occurred around 1 h, while the maximum inhibitory effects of NO, TNF-α, and IL-6 were observed after more than 10 h, suggesting a lag in pharmacological effects relative to drug concentration. The K_e0_ parameter for PTD suggests a moderate delay in modulating TNF-α, implicating TNF-α as a primary target for PTD’s anti-rheumatoid arthritis effects in vivo. IC50 comparisons revealed that TNF-α is the primary target for PTD. The PK-PD analysis of PTD highlights its significant potential as an anti-inflammatory agent in treating rheumatoid arthritis. The observed peak concentration around 1 h and the prolonged maximum inhibitory effects on NO, TNF-*α*, and IL-6 over 10 h indicate that PTD has a sustained pharmacological impact, despite the initial delay in effect.

### 2.7. PK-PD Analysis of PTD in Inflammatory Cell Model

**Figure 7:**
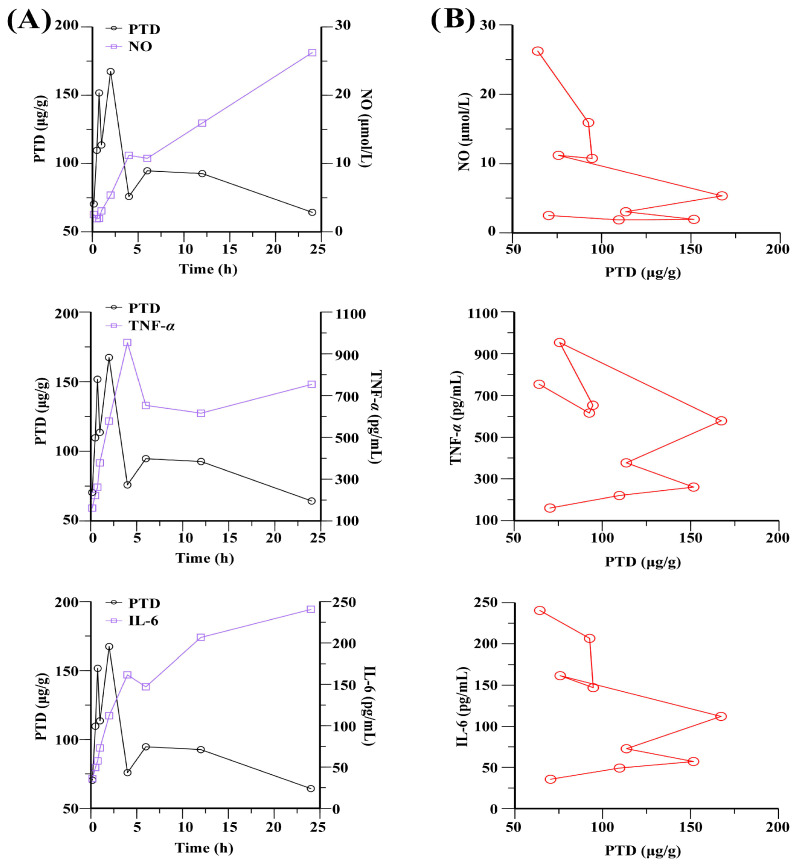
Concentration–time–effect curves (**A**) and concentration–effect curves (**B**) of PTD with NO, TNF-*α*, and IL-6 in inflammatory cells. The red lines represent the relationship between the drug concentration and the pharmacodynamic index concentration.

A two-compartment PK model and a PD model with an effect-site compartment were combined to establish an S-Imax PK-PD model with a high degree of fit, as illustrated in Figure 7. The model effectively describes the time–concentration–effect relationship. It was observed that the maximal inhibitory effects lagged behind the intracellular concentration peaks. The time–concentration–effect curves exhibited an anticlockwise hysteretic loop between the intracellular concentration of PTD and the levels of TNF-α, IL-6, and NO. Consequently, a PK-PD model with a separated effect compartment was introduced for data analysis. The PK-PD model parameters of PTD, calculated using WinNonLin 8.2 software, are summarized in Table 3. The PK-PD analysis of PTD reveals its significant potential as an anti-inflammatory agent. The establishment of the S-Imax PK-PD model with a high degree of fit effectively describes the time–concentration–effect relationship, highlighting the distinct hysteretic loops observed. The maximal inhibitory effects lagged behind the intracellular concentration peaks, indicating a time-dependent relationship between PTD concentrations and its pharmacodynamic impacts. The anticlockwise hysteretic loop observed between the PTD intracellular concentration and TNF-α, IL-6, and NO levels suggests a delayed but sustained effect on these inflammatory markers. This implies that PTD has a prolonged pharmacological action, which could be beneficial for managing chronic inflammatory conditions, such as rheumatoid arthritis. The PK-PD model parameters further emphasize the potential of PTD. The relatively high E_0_ value for TNF-*α* indicates a significant baseline level of these markers, while the high K_e0_ value for TNF-*α* suggests a rapid onset of effect. The γ and IC50 values provide insights into the potency and specificity of PTD, with lower IC50 values indicating greater efficacy.

## 3. Discussion

The findings reveal the promising therapeutic potential of BDE and PTD in both animal and cellular models of inflammation and autoimmune diseases. The histopathological improvements in the AA rat model, paired with the reduction in inflammatory markers, such as TNF-α and IL-6, suggest that BDE is effective in mitigating rheumatoid arthritis pathology through its anti-inflammatory and immunomodulatory effects. This aligns with the known roles of these cytokines in driving synovial inflammation and autoimmune responses, indicating that BDE may serve as a potent therapeutic agent. In the cellular model, the ability of BDE and PTD to significantly suppress LPS-induced inflammatory markers, including NO, TNF-α, and IL-6, demonstrates their efficacy in reducing cellular inflammation [15,16]. This anti-inflammatory effect suggests that these compounds not only modulate cytokine secretion but may also inhibit key pathways activated by LPS. The negligible cytotoxic effects of BDE and PTD further strengthen their potential as safe and effective therapeutic agents. LPS, a structural component of the outer membrane in Gram-negative bacteria, is frequently utilized in experimental research to elicit inflammatory reactions [17]. Upon contact with cells, especially immune cells, such as macrophages, LPS initiates a sequence of molecular events that culminate in the secretion of inflammatory markers, including NO, TNF-α, and IL-6 [18]. NO functions as a signaling molecule that participates in diverse physiological processes, including inflammation. TNF-α and IL-6 are cytokines that promote inflammation and are vital for the initiation and modulation of immune responses. Elevated levels of these markers signify that cells are effectively responding to LPS by activating inflammatory pathways. Thus, in a validated cellular model, increased concentrations of NO, TNF-α, and IL-6 would confirm the successful induction of inflammation by LPS, while a decrease in these markers would demonstrate the anti-inflammatory properties of BDE and PTD. The findings underscore the importance of exploring the pharmacokinetic and pharmacodynamic mechanisms underlying the anti-inflammatory effects of BDE and PTD. Their ability to simultaneously mitigate systemic and cellular inflammation highlights their versatility and potential in treating inflammatory and autoimmune diseases. Future studies should delve into their molecular interactions, particularly with cell surface receptors and intracellular pathways, to better understand their mechanisms of action and therapeutic applications.

The PK-PD analysis provides valuable insights into the mechanisms driving the anti-inflammatory effects of PTD in both AA rat models and LPS-induced inflammatory cell models. The data reveal the pharmacokinetic characteristics and the complex relationship between drug concentrations and pharmacodynamic responses, emphasizing the therapeutic potential of PTD in managing chronic inflammatory diseases, such as rheumatoid arthritis. In the AA rat model, the clockwise hysteretic loop observed indicates a delay between the peak plasma concentration of PTD and the modulation of inflammatory cytokines. This may be due to the time required for drug distribution to the target compartments or the pharmacological activity of its metabolites [19,20]. The IC50 and Ke0 parameters suggest that PTD targets key cytokines, including TNF-α, IL-1β, and RF, with TNF-α identified as the primary target [21]. The moderate delay in TNF-α modulation reflects a strong binding affinity and sustained pharmacological effect, highlighting PTD’s efficacy as an anti-inflammatory agent [22]. The reasons may be as follows: (a) Each drug efficacy index may belong to different effect compartments. The time required for the drug to be distributed from the central compartment to each effect compartment is different [23]. (b) The binding activity of the drug to the receptor is different [24]: high activity means a short lag time; low activity means a long lag time. The γ value plays a critical role in evaluating drug selectivity and sensitivity across a relevant concentration range [25]. Generally, a low in vivo γ (γ < 1) corresponds to a relatively flat pharmacodynamic profile, with minimal variation in the effect over a broad range of drug concentrations. At γ = 1, the relationship is typically represented by a hyperbolic model, while γ > 1 results in a sigmoid curve with a steeper slope. Minor concentration fluctuations near the ED50 can lead to effects ranging from negligible to nearly maximal. For high γ values (γ > 5), the concentration range required for a given effect is reduced to a simple threshold. In this study, the γ values for PTD in AA rats were found to be below 5, suggesting a relatively narrow margin of safety for BDE.

In the cellular model, the time-dependent pharmacodynamic impact of PTD was further demonstrated by the delay in the maximum inhibitory effects on NO, TNF-α, and IL-6. The prolonged pharmacological action observed, despite the initial delay, underscores the importance of intracellular drug concentrations and their critical role in eliciting anti-inflammatory effects. The parameters from the PK-PD analysis, such as IC50 and γ, emphasize the potency and specificity of PTD in modulating inflammatory pathways. Notably, the moderate γ values suggest that PTD has a relatively narrow margin of safety but maintains a stable pharmacodynamic profile within a therapeutic concentration range. The free drug concentration of PTD within the target cells is crucial for its pharmacological effects. The intracellular pharmacokinetics directly influence the drug’s efficacy, as evidenced by the prolonged inhibitory effects on NO, TNF-*α*, and IL-6. The delay between the peak drug concentration and the maximum inhibitory effects suggests a time-dependent relationship between the PTD concentration and its pharmacodynamic impact. This lag may be attributed to the time required for PTD to be distributed from the central compartment to the effect compartments and to bind to the target receptors. The IC50 comparisons and the K_e0_ parameter for PTD emphasize TNF-*α* as the primary target for its anti-inflammatory effects. The moderate delay in modulating TNF-*α* suggests that PTD has a strong binding affinity and a sustained impact on this cytokine, which is critical in the pathogenesis of rheumatoid arthritis. The sustained inhibitory effects of PTD on key inflammatory markers (NO, TNF-*α*, and IL-6) underscore its therapeutic potential in managing chronic inflammatory conditions like rheumatoid arthritis. The prolonged pharmacological action may translate to less frequent dosing regimens, improving patient compliance and treatment outcomes [26]. Overall, the PK-PD results for PTD provide valuable insights into its mechanism of action and highlight its potential as an effective treatment for rheumatoid arthritis. Further in vivo studies and clinical trials are warranted to confirm these findings. Additionally, there is a need for further PK-PD studies of PTD to optimize the dosing strategy for maximal therapeutic benefit.

The establishment of advanced PK-PD models, including the S-Imax model with a high degree of fit, further highlights the time–concentration–effect relationships and hysteretic loops observed in both intracellular and plasma contexts [27,28]. These findings reveal not only the sustained pharmacological action of PTD but also its time-dependent effects on key inflammatory markers. The counterclockwise hysteretic loops observed in the cellular models demonstrate a delayed but sustained inhibitory effect, suggesting potential benefits for the long-term management of inflammation. Overall, the PK-PD analyses of PTD in different models underscore its significant therapeutic potential, particularly its ability to modulate cytokine production and sustain anti-inflammatory effects [29]. These insights lay the groundwork for optimizing dosing strategies to maximize therapeutic efficacy while minimizing adverse effects. Further exploration in vivo and in clinical trials is essential to validate these findings and translate them into effective treatment protocols for chronic inflammatory conditions like rheumatoid arthritis.

While the PK-PD analyses of PTD provided significant insights into its anti-inflammatory effects and pharmacological characteristics, certain limitations should be noted. Firstly, the current study primarily focuses on preclinical models, which may not fully predict the pharmacokinetics and pharmacodynamics of PTD in human subjects. Translational studies and clinical trials are essential to validate these findings in a clinical setting. Secondly, the observed delays in pharmacological effects warrant further investigation into the drug’s distribution dynamics and interactions with specific receptors or intracellular pathways. A deeper understanding of these mechanisms may help optimize its efficacy and dosing regimens. Additionally, although the IC50 values and γ parameters suggest the potency and specificity of PTD, the relatively narrow margin of safety underscores the need for careful dose optimization to minimize potential side effects. Further PK-PD modeling at different dosing levels could provide more robust data for establishing safe therapeutic windows [30,31]. In future studies, we plan to expand our research by doing the following: (A) conducting in vivo studies in larger animal models and initiating clinical trials to confirm the therapeutic efficacy and safety of PTD in humans; (B) investigating the molecular mechanisms underlying PTD’s interaction with inflammatory pathways and cytokines, particularly its binding affinity to specific receptors; (C) exploring combinatory treatments of PTD with other therapeutic agents to evaluate synergistic effects and potentially enhance its therapeutic index; and (D) refining PK-PD models to include more intricate variables, such as metabolite profiles, tissue-specific drug distribution, and long-term pharmacological effects [32]. By addressing these limitations and pursuing the outlined plans, we aim to further elucidate the therapeutic potential of PTD and optimize its application for managing chronic inflammatory conditions like rheumatoid arthritis.

## 4. Materials and Methods

### 4.1. Drug and Reagents

*B. decapetala* (Batch. 20190823), acquired from Wandongqiao medicinal market in Guizhou, China, was identified as the dried root of *Biancaea decapetala* (Roth) O. Deg. by Associate Professor Chunhua Liu from the Department of Pharmacognosy at Guizhou Medical University. The specimens are archived in the Guizhou Provincial Key Laboratory of Pharmaceutical Preparations under the number Hm-Yunshi-gy-190824.

Protosappanoside D (Lot. 21072101) was obtained from Shanghai Shidande Standard Technical Service (Shanghai, China). Puerarin (Lot. 110752-201816) was sourced from the National Institutes for Food and Drug Control (Beijing, China). Complete Freund’s adjuvant (CFA, Lot. SLBF1289) was acquired from Sigma Aldrich (St. Louis, MO, USA). All reference compounds had a purity greater than 98%. Australian fetal bovine serum (FBS, Lot. 2254375CP), Dulbecco’s Modified Eagle Medium (DMEM, Lot. 8122356), double antibody (Lot. 15140-122) and tryptic digest (Lot. 25200-056) were purchased from Gibco (Carlsbad, CA, USA). LPS (Lot. L8880), and BCA Protein Assay Kit (Lot. 20230507) was obtained from Solarbio Science & Technology Co., Ltd. (Beijing, China). MTS (Lot. G358C) was purchased from Promega Biotech Co., Ltd. (Beijing, China). ELISA kits for TNF-*α* (Lot. 20221224), IL-6 (Lot. 20221214), IL-1*β* (Lot. 20221209), and NO (Lot. A013-2-1) were obtained from Nanjing Jiancheng Biotechnology Co., Ltd. (Nanjing, China). LC–MS/MS-grade methanol, formic acid, and acetonitrile were sourced from Merck Co., Ltd. (Darmstadt, Germany). Distilled water was obtained from Watsons Group Co., Ltd. (Hong Kong, China).

### 4.2. Preparation of B. Decapetala Extract

The dried roots of *B. decapetala* (30 kg) were extracted twice with 70% ethanol at ratios of 1:10 and 1:8 (*w*/*v*) for 1.5 h and 1.0 h, respectively. The combined extraction solutions were filtered and concentrated under reduced pressure to yield a crude extract with a concentration of 1 g/mL. The process produced a crude drug yield of 10.0% (g/g), with analyzed contents, including PTD, at 90.60 mg/g.

### 4.3. Solution Preparation

#### 4.3.1. Preparation of Internal Standard Solution

A specified amount of puerarin reference substance was weighed, dissolved in methanol in a 10 mL volumetric flask, and diluted to prepare a stock solution at 0.495 mg/L. This stock solution was further diluted in methanol to obtain internal standard working solutions at concentrations of 20 ng/mL and 5 ng/mL. All solutions were stored at −20 °C until use.

#### 4.3.2. Preparation of Stock Solutions and Quality Control Samples

PTD was accurately weighed and dissolved in methanol to prepare stock solutions at a concentration of 0.5021 mg/mL. For the rat PK study, the standard solution was spiked into blank plasma to prepare the standard curve and quality control (QC) samples. Similarly, in cellular PK experiments, the standard was diluted with blank cell lysates to generate the standard curve and QC samples.

#### 4.3.3. Preparation of LPS Solution

An appropriate amount of LPS (10 mg) was dissolved in 10 mL of PBS to prepare a stock solution at 1 mg/mL. This solution was filtered through a 0.22-μm membrane and stored at −20 °C. Prior to use, it was diluted to the required concentration with complete medium.

#### 4.3.4. *B. decapetala* Extracts Stock Solution

An appropriate amount of BDE was dissolved in 0.5% CMC-Na to prepare a 4.5 g/kg stock solution for rat pharmacokinetic experiments. For cellular pharmacokinetic experiments, approximately 0.80 g of BDE was dissolved in DMSO via ultrasonication, then diluted with PBS to a final volume of 25 mL, resulting in a concentration of 101.81 mg/mL. This solution was filtered through a 0.22–μm membrane and stored in aliquots at −20 °C. It was diluted with complete medium before use.

### 4.4. Sample Preparation for Plasma and Cell Lysates

For plasma sample preparation, 100 µL of plasma was mixed with 50 µL of IS (20 ng/mL puerarin), 50 µL of 2% formic acid, and 400 µL of methanol. The mixture was vortexed for 1 min, sonicated for 10 min, and centrifuged at 12,000 rpm for 10 min at 4 °C. The supernatant was evaporated to dryness under nitrogen at 37 °C, reconstituted in 150 µL of 50% methanol, and centrifuged again under the same conditions. For cell lysate preparation, 800 µL of lysate was combined with 50 µL of IS (5 ng/mL puerarin), 50 µL of 5% formic acid, and 800 µL of methanol. The mixture was vortexed for 5 min, sonicated for 10 min, and centrifuged at 12,000 rpm for 10 min at 4 °C. The supernatant was evaporated to dryness under nitrogen at 37 °C, reconstituted in 180 µL of 50% methanol, and centrifuged again. The final supernatants from both plasma and cell lysates were collected for UPLC-MS/MS analysis.

### 4.5. UPLC-MS/MS Conditions for Qualitative and Quantitative Analysis

Qualitative and quantitative analyses were performed using UPLC-MS/MS. For qualitative analysis, a Vanquish Horizon UPLC system coupled with a Q Exactive Plus Orbitrap HRMS Spectrometer (Thermo Fisher, Waltham, MA, USA) was used. The spectrometer operated in positive and negative modes with a spray voltage of 3.5 kV (positive) and 2.5 kV (negative), a capillary temperature of 300 °C, and a probe heater temperature of 350 °C. Full MS scans (resolution:70,000) and dd-MS^2^ scans (resolution: 17,500) covered *m*/*z* 100–1000. Separation was achieved using a Hypersil Gold column (2.1 × 150 mm, 1.9 μm) at 40 °C with a mobile phase of 0.1% formic acid in water (A) and 0.1% formic acid in acetonitrile (B), flowing at 0.3 mL/min. The gradient program was: 0–2 min (5% B), 2–20 min (5%~15% B), 20–30 min (15%~25% B), 30–40 min (25%~40% B), 40–50 min (40%~95% B), 50–52 min (95%~95% B), 52–53 min (95%~5% B), and 53–55 min (5% B).

For quantitative analysis, an ACQUITYTM UPLC system (Waters, Milford, MA, USA) with a BEH C18 column (100 × 2.1 mm, 1.7 μm) was employed. The mobile phase consisted of water (A) and acetonitrile (B), both containing 0.2% formic acid, at a flow rate of 0.30 mL/min. The gradient program was: 0–0.5 min (10% B), 0.5–3.0 min (10–90% B), 3.0–3.5 min (90% B), and 3.5–4 min (90–10% B). Detection was performed using a Waters XEVO TQ-S Triple-Quadrupole Mass Spectrometer with an ESI source. Key parameters included an ion source temperature of 120 °C, desolvation gas temperature of 350 °C, and capillary voltage of 3.0 kV. Quantification was conducted via MRM in positive and negative modes, with analyte and IS parameters detailed in Table S14. Data were processed using Micromass Masslynx V4.1 software.

### 4.6. Animal Experiment Design

Male Sprague–Dawley rats (180–220 g, 6–8 weeks) were obtained from Beijing HFK Bioscience Co., Ltd. (Certificate No. SCXK Jing 2019-0008, Beijing, China) and acclimatized for 1 week under controlled conditions (temperature: 20–25 °C, humidity: 55–65%). This study was approved by the Animal Care Welfare Committee of Guizhou Medical University (Ethics No. 1603125).

Twenty-four rats were divided into four groups (*n* = 6): normal control, model (AA), positive control (AA + POS), and *B. decapetala* extract (AA + BDE). Except for the control group, rats were immunized with CFA (0.1 mL) via intradermal injection on the right foot on days 1 and 7 to establish an AA model. The control group received saline injections. From day 21, the AA + BDE group was orally administered BDE (4.5 g/kg, twice daily), while the AA + POS group received tripterygium glycosides (102 mg/kg, twice daily) for 14 d. On the final day, 2 h post-administration, blood was collected from the tail vein, and plasma levels of TNF-*α*, IL-1*β*, IL-6, and rheumatoid factor (RF) were measured using ELISA kits. Rats were then euthanized, and the right ankle joint was excised. Soft tissues were removed, and joints were fixed in 4% paraformaldehyde for hematoxylin–eosin (HE) staining. Histopathological changes were observed under a microscope to evaluate the AA model’s stability and the therapeutic effects of BDE. HE scoring criteria are as follows: (1) Subsynovial inflammation: normal = 0 points; focal inflammatory cell infiltration = 1 point; diffuse inflammatory cell proliferation = 2 points. (2) Synovial hyperplasia: normal = 0 points; proliferation of synovial cells in more than three layers in one joint surface = 1 point; proliferation of synovial cells in more than three layers in multiple joint surfaces = 2 points. (3) Pannus formation and cartilage destruction: normal = 0 points; pannus covering the cartilage without cartilage destruction = 1 point; presence of pannus and cartilage destruction = 2 points.

### 4.7. Rat PK-PD Model

Six SD rats were subjected to the same modeling and administration procedures as the AA + BDE group described in Section 4.6. Blood samples (0.25 mL) were collected from the tail vein before administration and at 0.083, 0.167, 0.25, 0.5, 1, 2, 3, 4, 6, 8, 10, 12, and 24 h after the last dose. Blood loss was compensated by administering an equivalent volume of normal saline to maintain fluid balance. Plasma was separated by centrifugation at 6000 rpm for 10 min at 4 °C, and 200 μL aliquots were stored at −80 °C for PK and PD analyses.

The plasma concentrations of PTD, along with TNF-*α*, IL-1*β*, IL-6, and RF levels, were used to establish PK-PD relationships. Model fitness was evaluated using Akaike’s Information Criteria (AIC). The PK profile of BDE in AA rats was best described by a two-compartment model, and the Sigmoid Imax model was selected as the optimal PK-PD model based on its superior fit:

(1) $$E=E_{0}-\frac{I_{\max}\times C^{\gamma }}{{{\mathrm{IC}}_{50}}^{\gamma }+C^{\gamma }}$$

where E represents the effect value, C represents the mass concentration of the drug in the effect room, *γ* represents the midpoint slope of the curve, a shape coefficient that describes the sensitivity of the concentration–effect relationship, E_0_ represents the basic value of the drug effect, and IC_50_ refers to the inhibitory concentration when the inhibitory effect reached 50%, which reflects the affinity between the drug and the receptor. I_max_ represents the maximum effect of the drug, reflecting the intrinsic activity of the drug. The higher the value, the greater the intrinsic activity.

### 4.8. Cell Culture and Treatments

RAW264.7 mouse mononuclear macrophage cell line (Lot. CL-0910) was obtained from Wuhan Puno Life Sciences Co. (Wuhan, China). Cells were cultured in DMEM supplemented with 10% FBS at 37 °C and 5% CO_2_, with serial passaging at a 1:6 ratio. Upon reaching 90% confluency, cells were trypsinized, adjusted to a density of 1 × 10^5^ cells/mL, and seeded into 96-well plates at 100 μL per well for 24 h incubation.

For the inflammatory model, cells were treated with LPS at concentrations of 0.01, 0.025, 0.05, 0.1, 0.2, 0.5, 1.0, 2.0, or 5.0 μg/mL (six replicates per concentration). Cell viability was assessed using the MTS assay to determine the optimal LPS concentration. For drug treatment, cells were exposed to BDE (0–5000.0 µg/mL) or PTD (0–400 μmol/L) for 24 h. After incubation, cell viability was measured using the MTS assay, and the percentage viability was calculated as follows:

(2) $$\mathrm{Cell}\;\mathrm{viability}\;(\%)\;=\frac{\mathrm{OD}\;(\mathrm{experimental}\;\mathrm{group})}{\mathrm{OD}\;(\mathrm{blank}\;\mathrm{group})}\times 100\%$$

Cells were then divided into five groups (*n* = 6): normal control, model (AA), positive control (AA + POS, 100 μg/mL dexamethasone), BDE (800 µg/mL), and PTD (200 µmol/L). The levels of NO, TNF-*α*, and IL-6 in the supernatant were measured using ELISA to evaluate the anti-inflammatory effects of BDE and PTD.

### 4.9. Cellular PK-PD Model

RAW264.7 cells in the logarithmic growth phase were seeded at 1.5 × 10^5^ cells/mL in 6-well plates and incubated for 24 h to allow adherence. After washing with PBS, cells were treated with 2 mL of LPS (500 ng/mL) in complete culture medium (10% FBS, 1% penicillin–streptomycin, and 89% DMEM) for 24 h. The medium was then discarded, and cells were washed twice with PBS before adding 6 mL of BDE (800 µg/mL) or PTD (200 µmol/L) in serum-containing medium. Cells were incubated for 0.17, 0.5, 0.75, 1, 2, 4, 6, 12, and 24 h. After incubation, cells and supernatants were collected. Cells were washed three times with cold PBS, resuspended in 1 mL of cold ultrapure water, and subjected to three freeze–thaw cycles followed by 30 min of ultrasonication for complete lysis. Lysates were stored at −80 °C for PK analysis (BDE component concentrations), while supernatants were used for PD analysis (NO, TNF-α, and IL-6 levels). PK-PD fitting was performed as described in Section 4.7.

### 4.10. Data Analysis

PK-PD parameters were analyzed using WinNonLin software version 8.2 (Pharsight Corporation, Mountain View, CA, USA). Data are expressed as mean ± standard deviation. Statistical analyses were performed with SPSS 23.0 (IBM, Chicago, IL, USA), employing one-way ANOVA for intergroup comparisons. * *p* < 0.05 was considered statistically significant.

## 5. Conclusions

The PK-PD analysis of PTD underscores its significant potential as an effective anti-inflammatory agent. The distinct hysteretic loops and model parameters provide valuable insights into its mechanism of action. The sustained inhibitory effects on key inflammatory markers, coupled with a prolonged pharmacological action, suggest that PTD could be beneficial in managing chronic inflammatory conditions with improved dosing regimens and patient compliance. Further in vivo studies and clinical trials are warranted to confirm these findings and optimize the dosing strategy for maximal therapeutic benefit.

## Figures and Tables

**Table 1:** PK-PD parameters of PTD with TNF-*α*, IL-1*β*, IL-6, and RF as efficacy indicators in AA rats after administration of BDE.

PK Indicator	PD Indicators	Parameters			
		E_0_	K_e0_	γ	IC50
PTD	TNF-*α*	362.82	3.4 × 10^−4^	0.51	432.33
	IL-1*β*	241.01	0.34	0.04	956.05
	IL-6	152.80	0.05	1.78	874.91
	RF	54.14	0.37	2.03	5107.66

**Table 2:** PK-PD parameters of PTD with NO, TNF-*α*, and IL-6 as the efficacy indicators in inflammatory cells after administration of BDE.

PK Indicator	PD Indicators	Parameters			
		E_0_	K_e0_	γ	IC50
**PTD**	NO	16.12	4.79 × 10^−5^	0.08	20.98
	TNF-*α*	312.85	2.38 × 10^−7^	0.52	0.97
	IL-6	443.73	6.12 × 10^−8^	0.13	0.55

**Table 3:** PK-PD parameters of PTD with NO, TNF-*α*, and IL-6 as efficacy indicators in inflammatory cells.

PK Indicator	PD Indicators	Parameters			
		E_0_	K_e0_	γ	IC50
PTD	NO	26.78	3.5 × 10^−4^	2.83	103.42
	TNF-*α*	508.01	8.04	8.42	479.53
	IL-6	8.76	0.42	2.78	118.18

## Data Availability

The original contributions presented in this study are included in this article/Supplementary Material, and further inquiries can be directed to the corresponding authors.

## Supplementary Materials

The following supporting information can be downloaded at https://www.mdpi.com/article/10.3390/ijms26083694/s1.
