# Supplementary material for: A Pharmacokinetic–Pharmacodynamic Study of Protosappanoside D, a Component Derived from Biancaea decapetala Extracts, for Its Anti-Inflammatory Effects
Source: Int J Mol Sci. 2025 Apr 14;26(8):3694. doi: 10.3390/ijms26083694 (PMC12027796; doi:10.3390/ijms26083694)
Supplement: Supplementary file 1 [file ijms-26-03694-s001.zip › ijms-3511982-supplementary.pdf]

Supplementary Material

Figures:

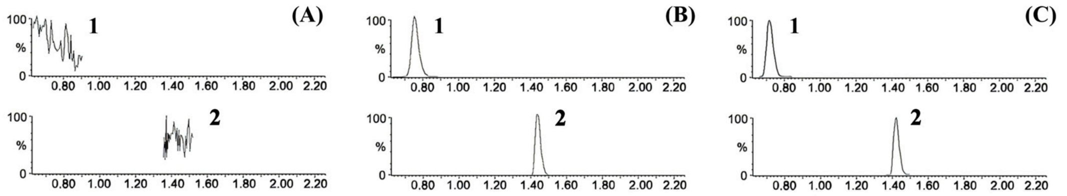

Figure S1. Typical UPLC-MS/MS chromatograms of blank plasma samples (A), blank plasma samples spiked with three analytes at LLOQ and puerarin (B), and actual plasma samples (C). 1. PTD; 2. Puerarin (IS).

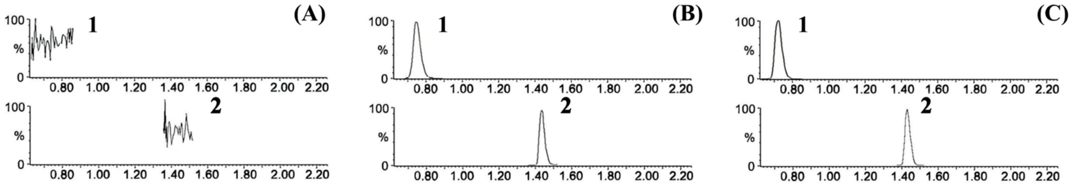

Figure S2. Typical UPLC-MS/MS chromatograms of blank cell lysates (A), blank cell lysates spiked with three analytes at LLOQ and puerarin (B), and actual cell lysates (C). 1. PTD; 2. Puerarin (IS).

Tables:

Table S1 Component identification *in vitro* and *in vivo* of BDE using UHPLC/Q Exactive Plus Orbitrap high-resolution mass spectrum.

No	Name	RT	Formula	MW	Adducts	MS[1]	Erro /ppm	MS[2]	Medicated plasma-RT
1	Gluconic acid	1.3	$C_6H_{12}O_7$	196.0584	$[M-H]^-$	195.0511	0.3	177.0404, 159.0295, 129.0188, 101.0236	1.2
2	L(-)-Carnitine	1.3	$C_7H_{15}NO_3$	161.1058	$[M+H]^+$	162.1131	3.7	103.0398, 102.0922, 85.0294	1.3
3	Betaine	1.3	$C_5H_{11}NO_2$	117.0797	$[M+H]^+$	118.0870	5.8	102 .0559, 71.0695, 59.0740	1.4
4	Trigonelline	1.3	$C_7H_7NO_2$	137.0482	$[M+H]^+$	138.0555	3.9	136.0400, 111.0448, 110.0609, 96.0453, 94.0660	
5	α,α-Trehalose	1.3	$C_{12}H_{22}O_{11}$	342.1176	$[M-H]^-$	341.1103	3.9	281.0893, 179.0561, 161.0453, 119.0343	
6	Quinic acid	1.3	$C_7H_{12}O_6$	192.0618	$[M-H]^-$	191.0545	-8.5	173.0455, 171.0299, 143.0338, 137.0288, 111.0080	
7	L-Pipecolic acid	1.4	$C_6H_{11}NO_2$	129.0796	$[M+H]^+$	130.0869	4.7	112.0767, 84.0817, 82.0664	1.4
8	Citric acid	1.7	$C_6H_8O_7$	192.0270	$[M-H]^-$	191.0197	-0.2	173.0089, 154.9981, 147.0296, 129.0187, 111.0079	
9	N-Acetylglutamate	1.7	$C_7H_{11}NO_5$	189.0644	$[M-H]^-$	188.0568	1.9	170.0458, 146.0456, 144.0662, 128.0347, 102.0553	
10	Citramalic acid	1.9	$C_5H_8O_5$	148.0367	$[M-H]^-$	147.0296	-2.2	129.0188, 103.0393, 87.0079, 85.0286	
11	Pyrogallol	2.0	$C_6H_6O_3$	126.0311	$[M-H]^-$	125.0239	-4.5	124.0160, 108.0209, 107.0132, 97.0288	2.0
12	Xanthurenic acid	3.3	$C_{10}H_7NO_4$	205.0384	$[M+H]^+$	206.0458	4.9	206.0457, 178.0506, 160.0405, 150.0555, 132 .0451	
13	Methylsuccinic acid	3.4	$C_5H_8O_4$	132.0418	$[M-H]^-$	131.0345	-3.8	113.0242, 87.0442, 85.0285	3.4
14	Protocatechuic acid	3.8	$C_7H_6O_4$	154.0264	$[M-H]^-$	153.0192	-0.8	152.0112, 110.0002, 109.0288	3.9
15	NP-020139	4.0	$C_{13}H_{16}O_9$	316.0813	$[M-H]^-$	315.0740	6.0	225.0414, 165.0187, 153.0190, 152.0111, 108.0209	

No	Name	RT	Formula	MW	Adducts	MS[1]	Erro /ppm	MS[2]	Medicated plasma-RT
16	Syringic acid	4.1	$C_9H_{10}O_5$	198.0536	$[M+H]^+$	199.0611	5.0	181.0504, 167.0347, 155.0710, 140.0475, 125.0241	
17	Protosappanoside D	5.8	$C_{22}H_{26}O_{11}$	466.1495	$[M-H]^-$	465.1422	4.1	303.0887, 231.0669, 185.0608, 159.0448	5.8
18	Catechin	7.7	$C_{15}H_{14}O_6$	290.0806	$[M-H]^-$	289.0736	6.3	245.0828, 205.0509, 203.0718, 151.0398, 125.0239	
19	Epicatechin	7.7	$C_{15}H_{14}O_6$	290.0804	$[M+H]^+$	291.0878	5.0	273.0764, 189.0552, 139.0396	
20	Vanillic acid	8.1	$C_8H_8O_4$	168.0427	$[M+H]^+$	169.0504	4.9	151.0397, 125.0604, 111.0449	8.1
21	Sinomenine	9.5	$C_{19}H_{23}NO_4$	329.1642	$[M+H]^+$	330.1715	4.5	239.0712, 223.0762, 181.0655	9.5
22	Pimelic acid	9.9	$C_7H_{12}O_4$	160.0733	$[M-H]^-$	159.0661	-1.4	141.0549, 115.0757, 97.0650, 95.0493	9.8
23	Paeonolide	10.0	$C_{20}H_{28}O_{12}$	460.1608	$[M-H]^-$	459.1535	5.8	165.0555, 150.0319, 71.0129	9.9
24	Brazilin	10.2	$C_{16}H_{14}O_5$	286.0856	$[M-H]^-$	285.0783	5.1	267.0677, 239.0725, 163.0400, 135.0447	10.2
25	(+)-3,3',4',5,7-Pentahydroxyflavan	10.9	$C_{15}H_{14}O_6$	290.0804	$[M+H]^+$	291.0873	3.5	165.0555, 147.0448, 139.0396, 123.0448	
26	Protosappanin B	12.1	$C_{16}H_{16}O_6$	304.0957	$[M-H]^-$	303.0892	5.8	243.0672, 231.0672, 213.055, 185.0598	12.1
27	Homobutein	12.2	$C_{16}H_{14}O_5$	286.0849	$[M+H]^+$	287.0920	2.2	270.0853, 243.0662, 242.0576, 226.0629, 211.0761	12.2
28	Magnoflorine	14.2	$C_{20}H_{23}NO_4$	341.1640	$[M+H]^+$	342.1715	4.5	297.1133, 265.0871, 237.0921	14.2
29	Citral	17.2	$C_{10}H_{16}O$	152.1208	$[M+H]^+$	153.1282	5.0	135.1175, 125.0604, 107.0864, 95.0865	
30	Suberic acid	17.5	$C_8H_{14}O_4$	174.0893	$[M-H]^-$	173.0820	0.3	155.0711, 129.0915, 111.0808, 109.0651	17.5
31	Prunin	20.3	$C_{21}H_{22}O_{10}$	434.1236	$[M-H]^-$	433.1177	8.4	272.0655, 271.0624, 151.0034, 119.0496	
32	Hematoxylin	21.9	$C_{16}H_{14}O_6$	302.0804	$[M-H]^-$	301.0742	8.2	283.0626, 257.0490, 179.0350, 151.03973	21.9
33	Cynaroside	22.2	$C_{21}H_{20}O_{11}$	448.1028	$[M+H]^+$	449.1097	4.0	287.0559, 241.0497, 213 0553, 153.0188, 121.0290	
34	Emodin	24.7	$C_{15}H_{10}O_5$	270.0541	$[M+H]^+$	271.0615	5.1	225 0559, 197.0608, 169 .0658	24.7

No	Name	RT	Formula	MW	Adducts	MS[1]	Erro /ppm	MS[2]	Medicated plasma-RT
35	Butein	25.0	$C_{15}H_{12}O_5$	272.0697	$[M+H]^+$	273.0766	3.2	255.0659, 237.0550, 163.0395, 137.0238, 107.0499	25.0
36	Kaempferol-7-O-glucoside	25.4	$C_{21}H_{20}O_{11}$	448.1028	$[M-H]^-$	447.0961	6.3	287.05591, 258.05304, 213.05533, 165.01892, 147.0449	
37	Azelaic acid	25.5	$C_9H_{16}O_4$	188.1051	$[M-H]^-$	187.0978	1.0	169.08694, 143.10744, 125.0966, 123.081	25.5
38	Syringaresinol	25.9	$C_{22}H_{26}O_8$	418.1645	$[M-H]^-$	417.1578	5.6	402.13361, 387.11017, 181.05052, 166.02687	
39	Kaempferol	25.9	$C_{15}H_{10}O_6$	286.0491	$[M+H]^+$	287.0564	4.7	241.05058, 213.05571, 185.06058, 137 .02406, 121.02921	25.9
40	Peonidine-3-O-glucoside	26.7	$C_{22}H_{22}O_{11}$	462.1186	$[M+H]^+$	463.1255	4.4	301.07193, 286.04846, 258 .05341	
41	Ligustilide	27.1	$C_{12}H_{14}O_2$	190.1002	$[M+H]^+$	191.1074	3.9	173.09706, 163.11252, 145.1019	
42	Phloridzin	28.0	$C_{21}H_{24}O_{10}$	436.1397	$[M-H]^-$	435.1325	6.4	273.07822, 229.08774, 179.03497, 167.03482	
43	Phloretin	28.0	$C_{15}H_{14}O_5$	274.0851	$[M+H]^+$	275.0927	4.8	169.05023, 151 03966, 149.06052, 107.04997	28.0
44	Daidzein	28.3	$C_{15}H_{10}O_4$	254.0589	$[M+H]^+$	255.0662	4.1	227.071, 199.07631, 181 06551, 137 .02388	28.3
45	Liquiritigenin	29.4	$C_{15}H_{12}O_4$	256.0751	$[M-H]^-$	255.0676	5.1	211.07652, 153.0191, 135.00832, 119.04962	29.4
46	Scrophulein	29.6	$C_{17}H_{14}O_6$	314.0805	$[M+H]^+$	315.0878	4.6	300.06412, 282.0535, 254.05844, 226.06326	
47	Glycitein	30.2	$C_{16}H_{12}O_5$	284.0701	$[M-H]^-$	283.0628	5.5	268.03879, 240.0435, 211.04033	30.2
48	Quercetin	30.9	$C_{15}H_{10}O_7$	302.0444	$[M-H]^-$	301.0373	6.3	273.0436, 257.0494, 178.9986, 151.0034, 121.0289	30.9
49	Luteolin	31.1	$C_{15}H_{10}O_6$	286.0492	$[M+H]^+$	287.0562	4.2	241.05064, 161.02406, 153.01895, 135.04474, 117.03439	31.0
50	Ethyl caffeate	31.9	$C_{11}H_{12}O_4$	208.0742	$[M-H]^-$	207.0669	2.9	179.03497, 161.02419, 134.03703, 135.04469, 133:02896	
51	3-Deoxysappanchalcone	32.9	$C_{16}H_{14}O_4$	270.0901	$[M+H]^+$	271.0978	4.6	253.16412, 225.05501, 145.10106	32.8

No	Name	RT	Formula	MW	Adducts	MS[1]	Erro /ppm	MS[2]	Medicated plasma-RT
52	9-hydroxy-2,10,10-trimethyltricyclo[6.3.0.0Âa,â◎µ]undec-6-ene-6-carboxylic acid	33.0	$C_{15}H_{22}O_3$	250.1581	$[M+H]^+$	251.1654	4.8	233.1547, 215.1441, 187.1490, 175.1126	
53	Qingyangshengenin	33.8	$C_{28}H_{36}O_8$	500.2442	$[M-H]^-$	499.2369	6.3	343.1932, 221.1191, 137.0240	33.8
54	Neochanin	34.1	$C_{16}H_{12}O_4$	268.0746	$[M+H]^+$	269.0821	4.5	254.0580, 213.0916, 193.0505	34.0
55	Apigenin	34.5	$C_{15}H_{10}O_5$	270.0542	$[M+H]^+$	271.0613	4.5	243.0663, 225 0555, 153.0189, 119.0499	34.5
56	Diosmetin	35.3	$C_{16}H_{12}O_6$	300.0647	$[M+H]^+$	301.0719	4.2	284.0341, 267.0320, 256.0388	35.3
57	6-Hydroxy-5a,9-dimethyl-3-methylene-3a,4,5,5a,6,7,9a,9b-octahydronaphtho[1,2-b]furan-2(3H)-one	35.4	$C_{15}H_{20}O_3$	248.1423	$[M+H]^+$	249.1497	4.6	231.1389, 213.1282, 195.118, 185.1333	
58	12-oxo Phytodienoic Acid	36.9	$C_{18}H_{28}O_3$	292.2052	$[M+H]^+$	293.2124	4.4	275.20181, 257.19125, 223.13388	36.9
59	Pimpinellin	37.0	$C_{13}H_{10}O_5$	246.0539	$[M+H]^+$	247.0612	4.5	232.0374, 231.0299, 215.0349, 203.0348, 87.0398	
60	lsoliquiritigenin	37.8	$C_{15}H_{12}O_4$	256.0747	$[M+H]^+$	257.0818	3.6	239.0711, 211.0761, 197.0801, 147.0447, 137 .0239	37.7
61	9-Oxo-10(E),12(E)-octadecadienoic acid	38.7	$C_{18}H_{30}O_3$	294.2203	$[M+H]^+$	295.2275	2.6	277.2172, 249.2222, 241.1959, 161.1332, 151.1124	
62	(+/-)12(13)-DiHOME	45.0	$C_{18}H_{34}O_4$	314.2471	$[M-H]^-$	313.2398	4.3	293.2151, 277.2184, 183.1391, 129.0915	
63	Hexadecanedioic acid	46.0	$C_{16}H_{30}O_4$	286.2158	$[M-H]^-$	285.2085	4.8	267.1979, 241.2180, 223.2073	

Note: RT means retention time; MW means molecular weight; MS[1] refers to primary fragment ions; MS[2] refers to secondary fragment ions.

Table S2 Calibration curves, linear ranges, correlation coefficients and LLOQ of PTD in rat plasma samples.

Analytes	Regression equation	R^2	Liner Range (ng/mL)	LLOQ (ng/mL)
PTD	Y= 0.0001X + 0.0175	0.9993	93.94 – 48096.00	93.94

Note: (1) Y, concerntration (ng/mL); X, Area. (2) LLOQ, Lower Limit of quantification.

Table S3 Precision and accuracy of PTD in rat plasma samples (n = 6).

Analyte	Theoretical concentration (ng/mL)	Intra-day			Inter-day		
		(Determined) ± SD (ng/mL)	Accuracy, RE (%)	Precision, RSD (%)	(Determined) ± SD (ng/mL)	Accuracy, RE (%)	Precision, RSD (%)
PTD	93.94	93.33 ± 7.74	-0.65	8.29	92.11 ± 6.16	-1.95	6.68
	187.88	191.52 ± 6.20	1.94	3.24	189.95 ± 6.51	1.10	3.42
	3006.00	2989.33 ± 80.57	-0.55	2.70	3128.47 ± 209.14	4.07	6.69
	24048.00	24195.93 ± 2089.11	0.62	8.63	23188.94 ± 2937.83	-3.57	12.67

Table S4 Recovery and matrix effect of PTD in rat plasma samples (n = 6).

Analyte	Theoretical concentration (ng/mL)	Recovery		Matrix effect	
		Mean ± SD (%)	RSD (%)	Mean ± SD (%)	RSD (%)
PTD	187.88	105.63 ± 3.92	3.92	100.48 ± 6.03	6.03
	3006.00	97.11 ± 5.37	5.37	95.98 ± 6.93	6.93
	24048.00	108.90 ± 5.76	5.76	97.25 ± 13.17	13.17

Table S5 Results of sample stability in plasma samples under different conditions (n = 6).

Analytes	Theoretical Concentration (ng/mL)	Room temperature for 24 h		4°C for 24 h		Three freeze-thaw cycles	
		Mean ± SD (%)	RSD (%)	Mean ± SD (%)	RSD (%)	Mean ± SD (%)	RSD (%)
PTD	187.88	191.90 ± 29.97	15.62	189.97 ± 15.61	8.22	180.92 ± 8.88	4.91
	3006.00	3079.47 ± 254.43	8.26	2824.01 ± 293.13	10.38	2917.28 ± 278.49	9.55
	24048.00	24947.13 ± 1096.84	4.40	24355.68 ± 1274.34	5.23	24875.32 ± 2099.86	8.44

Table S6 Calibration curves, linear ranges, correlation coefficients and LLOQ of PTD in cell lysate samples.

Analytes	Regression equation	R^2	Liner Range (ng/mL)	LLOQ (ng/mL)
PTD	Y = 0.006X - 0.0430	0.9999	10.00 – 1000.08	10.00

Note: (1) Y, concerntration (ng/mL); X, Area. (2) LLOQ, Lower Limit of quantification.

Table S7 Precision and accuracy of PTD in cell lysate samples (n = 6).

Analyte	Theoretical concentration (ng/mL)	Intra-day			Inter-day		
		(Determined) ± SD (ng/mL)	Accuracy, RE (%)	Precision, RSD (%)	(Determined) ± SD (ng/mL)	Accuracy, RE (%)	Precision, RSD (%)
PTD	10.00	10.32 ± 0.33	3.18	3.22	9.69 ± 0.37	-3.09	3.86
	25.00	23.40 ± 1.72	-6.41	7.36	25.82 ± 0.98	3.29	3.81
	125.00	135.47 ± 6.87	8.38	5.07	130.64 ± 3.16	4.51	2.42
	500.00	508.92 ± 37.05	1.78	7.28	515.46 ± 29.54	3.09	5.73

Table S8 Recovery and matrix effect of PTD in cell lysate samples (n = 6).

Analyte	Theoretical concentration (ng/mL)	Recovery		Matrix effect	
		Mean ± SD (%)	RSD (%)	Mean ± SD (%)	RSD (%)
PTD	25.00	99.23 ± 10.36	10.36	108.85 ± 7.07	7.07
	125.00	103.42 ± 1.06	1.06	101.13 ± 7.69	7.69
	500.00	102.91 ± 13.67	13.67	100.10 ± 10.64	10.64

Table S9 Results of sample stability in cell lysate samples under different conditions (n = 6).

Analytes	Theoretical Concentration (ng/mL)	Room temperature for 24 h		4°C for 24 h		Three freeze-thaw cycles	
		Mean ± SD (%)	RSD (%)	Mean ± SD (%)	RSD (%)	Mean ± SD (%)	RSD (%)
PTD	25.00	26.51 ± 0.85	3.21	25.77 ± 1.37	5.32	27.18 ± 1.01	3.71
	125.00	124.38 ± 5.28	4.25	120.62 ± 5.86	4.86	128.56 ± 5.05	3.93
	500.00	509.41 ± 25.28	4.96	488.78 ± 43.47	8.89	501.89 ± 36.32	7.24

Table S10 Time-dependent variation data of PTD and 3 PD indexes in the plasma of AA rats after oral BDE (Mean±SD, n = 6).

Time (h)	PTD (ng/mL)	TNF-α (pg/mL)	IL-1β (pg/mL)	IL-6 (pg/mL)	RF (pg/mL)
0.083	543.50±429.07	335.62±34.32	98.69±32.54	125.77±32.44	52.52±14.04
0.167	885.79±701.79	400.70±59.86	98.92±50.18	166.26±35.20	53.54±9.86
0.25	1343.27±747.29	235.05±47.60	103.44±57.69	150.84±35.44	49.42±9.87
0.5	1519.56±553.40	302.70±84.08	101.83±39.20	142.08±35.67	51.96±8.44
1	4094.63±1773.53	334.95±95.76	104.77±48.07	186.92±44.85	59.20±11.11
1.5	4150.12±1675.96	317.25±54.11	104.20±54.02	141.38±41.65	59.94±11.05
2	8756.71±6855.16	295.04±43.48	100.03±59.12	132.29±34.17	43.66±8.67
4	2508.10±1997.53	277.89±79.23	98.50±50.27	131.73±14.73	47.08±6.60

6	741.50±442.16	267.82±69.28	110.92±45.90	83.47±5.10	44.02±11.76
8	408.14±307.75	263.33±63.76	129.11±30.69	115.09±18.24	53.34±12.71
10	408.57±291.86	245.84±57.13	103.89±49.36	98.55±13.09	43.95±7.92
12	301.98±295.38	303.92±85.77	97.77±44.66	104.05±12.70	59.10±13.88

Table S11 Pharmacokinetic parameters of PTD in AA rats after oral BDE (Mean±SD, n = 6).

Pharmacokinetic parameters		PTD
$t_{1/2}$	h	6.19±2.74
T_{max}	h	1.29±0.33
C_{max}	ng/mL	9028.16±6577.25
$AUC_{(0-t)}$	h*ng/mL	12485.28±6180.59
$AUC_{(0-\infty)}$	h*μg/L	14081.32±5361.99
Vz/F	L/kg	2.90±1.88
CLz/F	L/h/kg	0.22±0.11
$MRT_{(0-\infty)}$	h	4.88±2.16

Table S12 Time-dependent variation data of PTD and 3 PD indexes in inflammatory cells (Mean±SD, n = 5).

Time (h)	PTD (ng/mL)	NO (μmol/L)	TNF-α (pg/mL)	IL-6 (pg/mL)
0.17	8.69±1.61	2.32±0.64	177.23±31.75	-
0.5	8.62±1.15	2.30±0.54	290.36±46.52	31.862±6.06
0.75	7.13±3.11	2.48±0.59	249.85±94.85	34.205±4.98
1	11.78±4.71	2.73±0.69	550.14±82.88	73.318±5.86
2	11.32±2.14	4.32±0.32	712.32±61.47	137.240±23.28
4	7.69±1.40	4.18±0.09	714.54±190.62	185.139±6.26
6	6.32±1.90	5.57±0.27	834.91±319.62	196.906±10.05
12	5.09±1.52	9.71±0.26	1002.87±179.59	407.822±17.35
24	5.30±0.42	14.46±1.60	1674.01±134.78	438.790±28.28

Note: '-': the detected concentration was below the quantification limit.

Table S13 Pharmacokinetic parameters of PTD in RAW264.7 cells after administration of BDE (Mean±SD, n = 5).

Pharmacokinetic parameters		PTD
$t_{1/2}$	h	27.76 ± 3.17
T_{max}	h	1.60 ± 0.55
C_{max}	ng/L	13.56 ± 3.62
$AUC_{(0-t)}$	h* ng/L	149.09 ± 22.89

$AUC_{(0-\infty)}$	h* ng/L	489.09 ± 269.04
V_d/F	mL	324.06 ± 79.23
Cl_z/F	mL/h	6.36 ± 2.05
$MRT_{(0-t)}$	h	10.58 ± 0.47
$MRT_{(0-\infty)}$	h	66.77 ± 56.06

Table S14 Mass spectrometric conditions of PTD and IS.

Analytes	Parent (*m/z*)	Daughter (*m/z*)	Cone (V)	Collision (V)	ESI
PTD	465.13	303.08	35	20	−
Puerarin (IS)	417.00	267.00	30	10	+
